# Determination of Bacteriocin Genes and Antibacterial Activity of Lactobacillus Strains Isolated from Fecal of Healthy Individuals

**Published:** 2017-02-28

**Authors:** Meysam Hasannejad Bibalan, Morteza Eshaghi, Mahdi Rohani, Mohammad Reza Pourshafie, Malihe Talebi

**Affiliations:** 1 *Department of Microbiology, Faculty of Medicine, Iran University of Medical Sciences, Tehran, Iran.*; 2 *Department of Microbiology, Pasteur Institute of Iran, Tehran, Iran.*

**Keywords:** Lactobacillus, bacteriocin genes, antibacterial activity, healthy individual

## Abstract

*Lactobacillus* species play a major role in gastrointestinal (GI) tract function, intestinal microbiota balance, and the immune system activity by exerting a strong activity against many intestinal pathogens. The aim of this study was to isolate *Lactobacillus* species from fecal samples, investigate their antimicrobial properties, and characterize their bacteriocin encoding genes. 48 fecal samples were grown in MRS broth and then MRS agar. The colonies grown in MRS agar were selected and identiﬁed by PCR. 72 *Lactobacillus* species were obtained from 434 lactic acid bacteria (LAB) strains. Approximately 40% of all *Lactobacillus* isolates had antimicrobial activity against one or more microorganisms and 17.4% of them were active against all four indicator bacteria. The frequency of bacteriocin encoding genes were 5 (6.9%), 3 (4.1%) and 5 (6.9%) for Gassericin A, Plantaricin S and Laf operon, respectively. pH alteration had no effect on antibacterial activity, but in the alkaline range these activities were reduced and the strains showed the highest antibacterial activity after 48 h incubation. These data indicate that the majority of isolates were susceptible to GI tract or belonged to other bacterial forms such as viable but nonculturable (VBNC). The detection of bacteriocin encoding genes in about only 6% of all *Lactobacillus* isolates seems to be due to the existence of many other bacteriocin encoding genes in *Lactobacillus* species which were not tested. Further study of the bacteriocin gene clusters, types, subtypes and the probiotic effect of these strains will contribute to a better characterization of these isolates.

Lactic acid bacteria (LAB) are a group of Gram-positive bacteria that produce lactic acid in two pathways: homofermentative or heterofermentative. These bacteria are found in nature, oral cavity, skin and human digestive system (1).

Lactobacillus species play an important role in gastrointestinal (GI) tract function, intestinal microbiota balance and the immune system activity by exerting a strong activity against many intestinal pathogens. These activities arise from the production of some specific components such as hydrogen peroxide, organic acids, inhibitory enzymes and bacteriocins (2).

In recent years, bacteriocins have attracted significant attention because of their potential use as safe additives for food preservation (3) and other applications such as treatment of pathogen diseases, cancer therapy and human health enhancement (4).

Bacteriocins are a heterogeneous group of ribosomally-synthesized pore-forming antibacterial peptides. These antibacterial peptides can be produced by many labs such as *Leuconostocetc*, *Streptococcus*,* Lactococcus*, *Pediococcus*, and* Lactobacillus* that enable these bacteria to inhibit the growth and activity of several pathogenic bacteria (5).

Lactobacilli have many different bacteriocins with the same activity but vary in some aspects such as mode of action, genetic origin (chromosomal or plasmid) and biochemical properties.These peptides are being tested to assess their application as narrow-spectrum antibiotics and nowadays, play an effective role in processing and packaging food and have major potential as natural preservatives (6, 7).

Based on killing mechanism, molecular weight, genetics and chemical properties, bacteriocins are classified in one of the four major groups: Class I (small peptide inhibitors termed as lantibiotics), Class II (small heat-stable proteins that have ﬁve subclasses), Class III (large, heat-labile protein and this class have two subclasses) and Class IV bacteriocins that are deﬁned as complex bacteriocins containing lipid or carbohydrate sections (8).

Lactobacilli usually produce the highest volume of bacteriocins during the maximum cell growth, but some environmental factors such as pH, temperature, incubation period and media composition can influence on the volume of bacteriocin production (9).

In this study, *Lactobacillus* species were isolated from fecal samples and their antimicrobial activities against pathogen microorganisms were determined. Moreover the genes encoding bacteriocins were investigated while the antibacterial activity was partially characterized.

## Materials and methods

**Bacterial strains and growth conditions**

Fecal samples were collected from 48 healthy volunteers who had no antibiotic therapy over 6 months before sampling and did not have GI disorders at the sampling time in Tehran, Iran. Samples were grown on Man, Rogosa and Sharpe (MRS) broth and then MRS agar (Merck, Germany) and incubated at 37 C for 48 h. Finally, the colonies were selected and kept at -80°C in MRS broth with 20% glycerol.

**Molecular identification of** ***Lactobacillus*** **isolates**

DNA was extracted using Bacterial Genomic DNA Purification Kit (Roche, Germany) according to the manufacturer's instruction. Identification of the *Lactobacillus* genus was carried out by PCR by using 16S rRNA specific For-Lac (5´-TGGAAACAGGTGCTAATACCG-3´) and Rev-Lac (5´-CCATTGTGGAAGATTCCC-3´) primers. PCR conditions were: 94 °C for 5 min; 30 cycles of 94 °C for 30 s, 57 °C for 30 s and 72 °C for 30 s, and a final extension step at 72 °C for 7 min (10).

**Screening for antibacterial** **activity**

**Agar spot tes**t 

The antibacterial activity of the isolates was determined by agar spot test according to Hernández et al. (11). Briefly, the strains were cultured in 5 ml of MRS broth at 37 ° C for 18 h and then, 3 μl aliquots of the culture were spotted onto agar plates containing 10 ml of MRS medium. After 18 h at 37 °C, the plates were overlaid with 5 ml of the appropriate soft agar (1 % agar) inoculated with the indicator strain (enteropathogenic* E. coli *(EPEC) (ATCC 4388), enteroaggregative* E. coli *(EAEC) (from Pasteur Institute of Iran and were not type strains), *Salmonella typhi *(ATCC 19430) and* Shigella dysenteriae* (PTCC 1188) with 10^5^ CFU /ml concentration. Finally, the plates were incubated at 37 °C for 24-72 h depending on the growth of the indicator strain and the inhibitory zone wider than 4 mm in diameter was considered as positive.

**Well-diffusion** **method**

The antibacterial activity of the isolates was determined by well-diffusion test according to Toba et al. (12). Briefly, the strains were cultured in 5 ml of MRS broth at 37 °C for 18 h and then, the cells were removed by centrifugation at 5000 rpm for 10 min. Wells were made on agar plates containing indicator strain (concentration equivalent to 10^5^ CFU/ml) and each well was inoculated with 100 µl (or less) of culture supernatant of *Lactobacillus* strains after neutralization with NaOH. Eventually, after an incubation period (37 °C for 24 h), the inhibitory zone wider than 6 mm in diameter was considered as positive.

**Detection of the bacteriocin genes**

PCR assay was used to determine bacteriocin genes, including: Gassericin A, Plantaricin S (structural gene) and Laf operon. PCR primers and annealing temperatures are shown in Table 1. PCR conditions were similar to all the genes: an initial denaturation step of 95 C for 5 min, 34 cycles of 95 C for 1 min, extension 72 C for 45 s, followed by a final extension at 72 C for 5 min (13). The amplified products were visualized by electrophoresis in 1.5% agarose gels stained with GelRed.

**Partial characterization of the antibacterial activity**

To study the effect of pH (native and ranging from 4 to 10) on antibacterial properties, the cell-free supernatant of lactobacilli culture was divided into two parts: one with native pH (pH= 4.7 resulting from bacterial growth on broth medium) and another with altered pH (pH= 4 to 10). After these treatments, the residual antibacterial activity was determined by the agar spot test and all experiments were carried out in duplicate (1).

To study the effect of incubation time on bacteriocin production, the equal fresh culture of *Lactobacillus* strains were incubated at 37 C for 17, 24, 48 and 72 h. At the end of each incubation time, the residual antibacterial activity was determined by the agar spot test and all experiments were carried out in duplicate (11).

## Results

**Identification of fecal isolates**

434 LAB strains were isolated from volunteers with healthy fecal samples. Among these isolates, 72 (16.5%) were identified as *Lactobacillus *species based on 16S rRNA gene amplification.

**Screening for** **antibacterial** **activity**

All 72 *Lactobacillus* species were assayed for potential bacteriocin production and their antimicrobial activity by using the agar spot test and well- diffusion method. The inhibitory activity of lactobacilli strains against indicator bacteria is reported in Table 2.

**Table 1:** Primers for bacteriocin genes

**Ref.**	**Amplicon size (bp)**	**Annealing temp (C)**	**Sequence (5′–3′)**	**Primer**	**Bacteriocin gene**
(6)	800	62	GAACAGGTGCACTAATCGGT	gas F	Gassericin A
			CAGCTAAGTTAGAAGGGGCT	gas R	
(13)	184	52	AGTCGTTGTTGGTGGAAGAAAT	F	Laf
			TCTTATCTTGCCAAAACCACCT	R	
(7)	320	60	GCCTTACCAGCGTAATGCCC	pln F	Plantaricin S
			CTGGTGATGCAATCGTTAGTTT	pln R	

**Table 2:** Determination of Lactobacillus antibacterial activity

**Indicator bacteria**	**Agar spot test N(%)**	**Well- diffusion method N(%)**
Enteropathogenic *E. coli* (EPEC)	34 (47.2)	37 (51.3)
Enteroaggregative *E. coli* (EAEC)	32 (44.4)	35 (48.6)
*Salmonella typhi*	28 (38.8)	27 (37.5)
*Shigella dysenteriae*	24 (33.3)	30 (41.6)

**Fig. 1:**
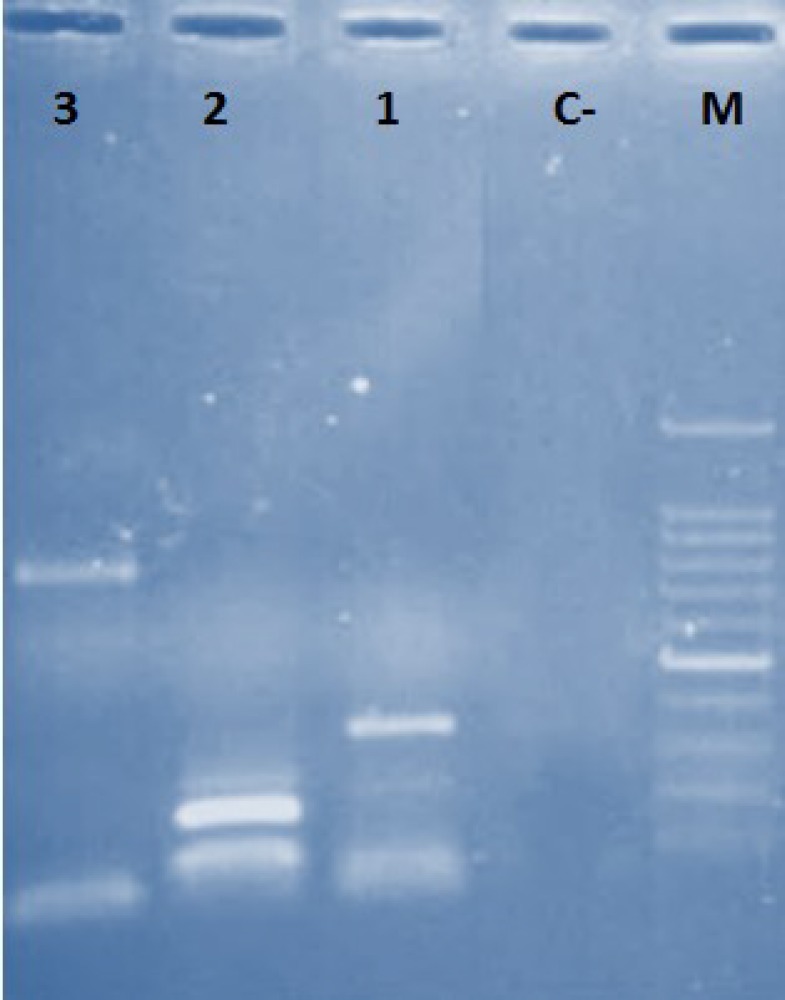
PCR product of *Lactobacillus* bacteriocin genes. M: 100 bp DNA ladder; C-: negative control; lane 1 through 3 represent Pln, Laf and Gas genes, respectively

**Detection of the bacteriocin genes**

All *Lactobacillus* species were tested for the presence of bacteriocin genes (Gassericin A, Plantaricin S and Laf operon). The frequency of these genes were 5 (6.9%), 3 (4.1%) and 5 (6.9%) for Gassericin A, Plantaricin S (structural gene) and Laf operon, respectively (Figure 1). We did not identify strains having at least two of the three tested genes.

**Partial characterization of the antibacterial activities**

pH alteration between 4 to 7 had no effect on antibacterial activities which arose due to the presence of bacteriocin components, but in the alkaline range (8 to 10), these activities were reduced. Regarding the effect of incubation time, the results showed that after 48 h incubation, the strains showed higher antibacterial activity in comparison with other incubation times tested.

## Discussion

In the present study, 72 (16.5%) *Lactobacillus* species were identified from a total of 434 LAB strains based on 16S rRNA gene amplification. Results showed that the large part of LAB isolates did not correspond to *Lactobacillus*. It may be possible that those species were more sensitive to GI tract in comparison with other LAB strains, or some other bacterial forms such as viable but nonculturable (VBNC) strains were present among the studied samples.

Our results showed that approximately 40% of all *Lactobacillus* isolates had antimicrobial activity against one or more microorganisms. Among these strains, 17.4% were active against all four indicator bacteria. These results are similar to other studies (6, 14, 15) about lactobacilli activity against a wide range of Gram-positive, and Gram-negative bacteria, although others reported different results with 10-14.3% of the isolates being able to inhibit the indicator organisms (1, 16).

The investigation of bacteriocin genes, including Gassericin A, Plantaricin S and Laf operon genes revealed a low frequency of bacteriocin genes between *Lactobacillus* strains. These results are similar to other studies (6, 13). We found that approximately 40% of all *Lactobacillus* isolates were positive for bacteriocin phenotypic test (antibacterial activity), but only 6% of all isolates had bacteriocin genes. This difference may be due to the existence of many different bacteriocin genes in *Lactobacillus* species.

Some environmental factors such as pH, temperature, incubation period and media composition can impact on the volume of bacteriocin production. Our results showed that pH alteration between 4 to 7 had no effect on antibacterial activity and these activities arose from bacteriocin components, but in the alkaline range (8 to 10), these activities were reduced. These results were similar to Mohankumar et al (1), but Ranveer et al. (5) and Kim et al. (9) stated that *Lactobacillus* species had their highest antibacterial activity at pH 7, whereas a considerable decrease was observed at both acidic and alkaline pH.

Regarding incubation time, our results showed that after 48 h incubation, the strains showed the highest antibacterial activity in comparison with other incubation times. Mohankumar et al. (1) and Ranveer et al. (5) obtained a maximum antibacterial activity after a 48 h incubation period. Some other studies were performed at different pH and temperature on bacteriocin production and activity (17, 18).

The study of the bacteriocin gene clusters, types, subtypes and the probiotic effect of these strains on laboratory animals will help to a better characterization of lactobacilli isolates.
